# Supplementary material for: Potent and Broad-Spectrum Bactericidal Activity of a Nanotechnologically Manipulated Novel Pyrazole
Source: Biomedicines. 2022 Apr 15;10(4):907. doi: 10.3390/biomedicines10040907 (PMC9029483; doi:10.3390/biomedicines10040907)
Supplement: Supplementary file 1 [file biomedicines-10-00907-s001.zip › biomedicines-1673277-supplementary.pdf]

Supplementary Materials

Strong Broad-Spectrum Bactericidal Activity of a Water Soluble Nanotechnologically Manipulated Novel Pyrazole

Silvana Alfei [1,*], Debora Caviglia [2], Alessia Zorzoli [3], Danilo Marimpietri [3], Andrea Spallarossa [1], Lusardi Matteo [1], Guendalina Zuccari [1] and Anna Maria Schito [2]

[1] Department of Pharmacy (DIFAR), University of Genoa, Viale Cembrano, 16148 Genoa, Italy; spallarossa@difar.unige.it (A.S.), zuccari@difar.unige.it (G.Z.), matteo.lusardi@edu.unige.it (M.L.)
[2] Department of Surgical Sciences and Integrated Diagnostics (DISC), University of Genoa, Viale Benedetto XV, 6, 16132 Genoa, Italy; amschito@unige.it (A.M.S.), debora.caviglia@edu.unige.it (D.C)
[3] Cell Factory Laboratory and Cell Therapy Center, IRCCS Istituto Giannina Gaslini, via Gerolamo Gaslini 5, 16147 Genoa, Italy; alessiazorzoli@gaslini.org (A.Z.), danilomarimpietri@gaslini.org (D.M.)
*Correspondence: alfei@difar.unige.it; Tel.: +39 010 355 2296 (S.A.)

Section S1. Synthesis and Characterization of CR232-loaded Dendrimer Nanoparticles (CR232-G5K NPs) [1].

S1.1. Preparation and Spectroscopic Characterization of CR232-Loaded Dendrimer NPs (CR232-G5K NPs)

The CR232-G5K NPs were prepared by modifying and merging the widely reported solution casting method and the solvent diffusion evaporation method [2]. G5K dendrimer (Gen 5.0) was dissolved in 1 mL of MeOH (pH = 7.4). To the dendrimer methanol solution, a strong excess of CR232 (42.3 equiv.) and MeOH (1 mL), were added obtaining a suspension which was incubated for 3 hours at 37 °C under vigorous stirring. Following incubation, the insoluble residue was separated, while the solution was evaporated. The evaporation of the alcoholic solution was performed at 70°C and at reduced pressure. The solid residue was suspended in 25 mL of water milli-Q, stirred for half an hour, and subsequently filtered. The residue was added to the first solid separated by the methanol solution and resuspended in methanol to dissolve all the subitizable. The solution was centrifugated (3500 rpm, 15′), separated by the residue, added to the water solution, and evaporated at reduced pressure. The thin film obtained was washed several times with acetone to remove residual water, subsequently with diethyl ether (Et_2O), brought to constant weight at vacuum, and stored in a dryer on P_2O_5 for further experiments (123.2 mg). The yellow washings containing the not entrapped CR232 were added to the previously separated residues to collect all not entrapped CR232 and were evaporated obtaining a yellow powder. The recovered crude CR232 was recrystallized by DCM/petrol ether and was obtained as yellow crystals. Both ATR-FTIR analysis (not reported spectrum) and TLC (DCM/MeOH 9/1), confirmed the identity of the recovered solid as CR232.

Spectroscopic Characterization of CR232-G5K NPs

ATR-FTIR (ν, cm^{-1}): 3500-3000 (NH_3^+ dendrimer), 3331 (NH CR232), 2935 (alkyl groups of the dendrimer), 2226 (CN of CR232) 1735 (C=O stretching esters of dendrimer), 1602 (CH=CH stretching phenyl rings of CR232), 1508, 1376 (NO_2 group of CR232) 1219, 1044 (C-O stretching esters of dendrimer).

^{1}H NMR (DMSO-*d6*, 400 MHz): δ = < 1 (CH_3 *core* not detected), 1.03-1.40 (m, 279 H, CH_3 of G1, G2, G3, G4, G5), 1.50-1.99 (m, 576 H, $CH_2CH_2CH_2$ of lys), 2.76 (m, 192 H, $CH_2NH_3^+$ of lys), 3.99 (m, 96 H, $CHNH_3^+$ of lys), 4.10-4.50 (m, 378 H, CH_2O of dendrimer), 7.61-7.67 (m, 82H, CH= aromatic ring), 7.68-7.70 (m, 82H, CH= aromatic ring), 7.84-7.86 (m, 82H, CH= aromatic ring), 8.16-8.18 (m, 82H, CH= of aromatic ring), 8.20 (br s, 288 H, NH_3^+), 8.82 (br s, 288 H, NH_3^+), 9.92 (bs, 41H, NH aniline, exchangeable with D_2O), 13.87 (bs, 41H, H pyrazole, exchangeable with D_2O). ^{13}C NMR (DMSO-*d6*, 100 MHz): δ = 19.33 (CH_3), 23.14 (CH_2), 28.01 (CH_2), 31.01 (CH_2), 40.02 ($CH_2NH_3^+$), 47.70 (quaternary C), 53.55 ($CHNH_3^+$), 67.65-67.82 (CH_2O and of G1, G2, G3, G4), 84.35, 119.49, 120.62, 130.81, 133.51, 134.78, 140.38, 144.61, 153.86, 170.68-173.33 (C=O of amino acid + ester of G1, G2, G3, G4). CH_3, quaternary C and CH_2O of *core* of G5K, as well as very small signals at 152.20 and 157.15 of CR232 were no detectable, one signal of pyrazole was overlapped. From ^{1}H NMR analysis: $C_{1702}H_{2510}N_{397}O_{460}Cl_{233}$; MW = 44153.1.

UV-Vis (DMSO): Peak of absorbance at λ_{abs} = 328 nm.

S1.2. Morphology and Average Size of G5K and CR232-G5K Particles

The morphology of the empty dendrimer G5K and of CR232-G5K particles was investigated by scanning electron microscopy (SEM) (Figure S1). In the performed experiments, samples were fixed on aluminum pin stubs and sputter-coated with a gold layer of 30 mA for 1 min, and an accelerating voltage of 20 kV was used for the sample's examination. The micrographs were recorded digitally using the DISS 5 digital image acquisition system (Point Electronic GmbH, Halle, Germany).

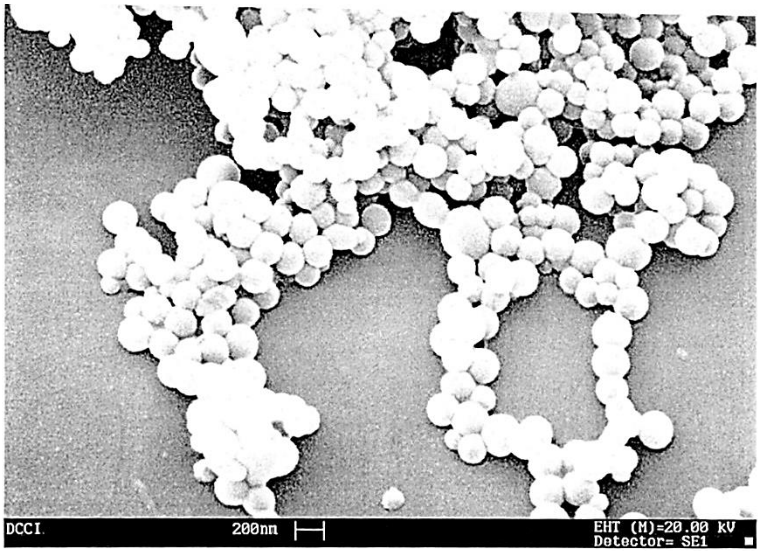

(**a**)

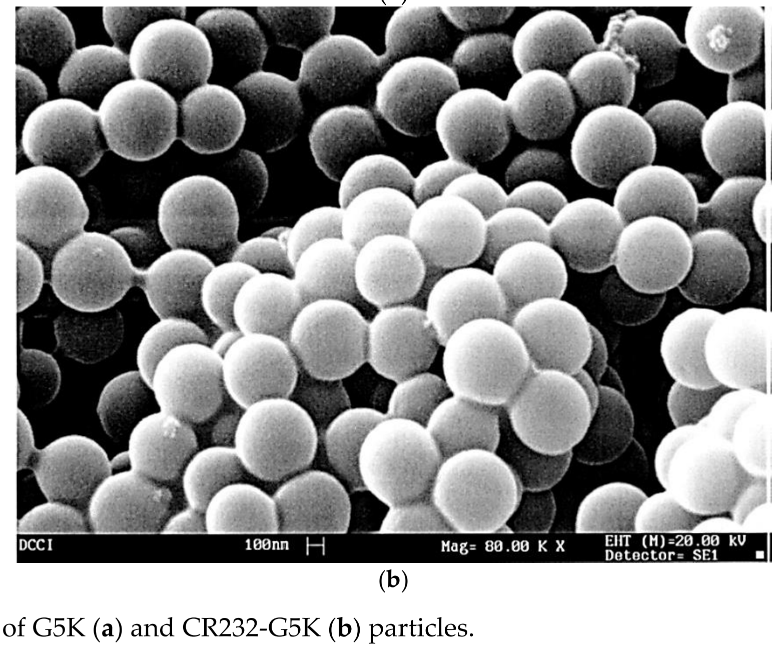

(**b**)

Figure S1. SEM images of G5K (**a**) and CR232-G5K (**b**) particles.

S1.3. Content of CR232 in CR232-G5K NPs, Drug Loading (DL%) and Encapsulation Efficiency (EE%)

S1.3.1. CR232 calibration curve

A stock solution of CR232 (0.1 mg/mL) was prepared in DMSO, by diluting an initial solution obtained dissolving 5 mg of CR232 in 50 mL of DMSO. Dilutions with DMSO were made, to prepare standard solutions at concentrations of 0.02494, 0.01995, 0.01496, 0.00998, 0.00499 mg/mL. The content of CR232 present in each solution

was quantified using the UV-Vis apparatus described in the previous Section 2.7 by detecting the absorbance (A), at room temperature and at λ_{abs} = 384 nm. The CR232 calibration curve was obtained by the least-squares linear regression analysis of the CR232 concentrations vs. the A signals created in the UV detector by the different concentrations of the analyte (CR232). Determinations were made in triplicate and the A values obtained for each CR232 concentration analysed, were expressed as mean ± SD (A_{mean} ± SD). The equation of the developed linear calibration model was the following Eq. (1).

$$y = 60.019\,x + 0.0107 \quad (1)$$

where y was the A values measured at λ_{abs} = 384 nm and x the CR232 standard concentrations analysed. In Eq. (1) the slope represents the coefficient of extinction (ε) of CR232. Table S1 collects the values of A (expressed as A_{mean}) determined for each CR232 concentration injected in the UV-Vis system, the concentrations of CR232 (C_{CR232}) used for the UV-Vis analyses, the CR232 concentrations predicted by the calibration model (C_{CR232p}), the residuals and the absolute errors percentages.

Table S1. Data of the calibration curve: A_{mean}, C_{CR232}, C_{CR232p}, residuals, and absolute errors (%).

C_{CR232} (mg/mL)	A (mAU)	C_{CR232p} (mg/mL)	Residuals (mg/mL)	Absolute errors (%)
0.02414	1.51560	0.02507	+0.00093	4.2
0.019952	1.19970	0.019181	-0.0001417	3.9
0.014964	0.88272	0.014529	-0.000435	2.9
0.009976	0.65521	0.01073	+0.000754	7.6
0.004988	0.29096	0.00468	-0.000308	6.2

A_{mean} and C_{CR232} reported in Table S1 were used to develop the CR232 calibration model by least squares method whose equation was Eq. (1). Figure S2 shows the obtained linear regression curve.

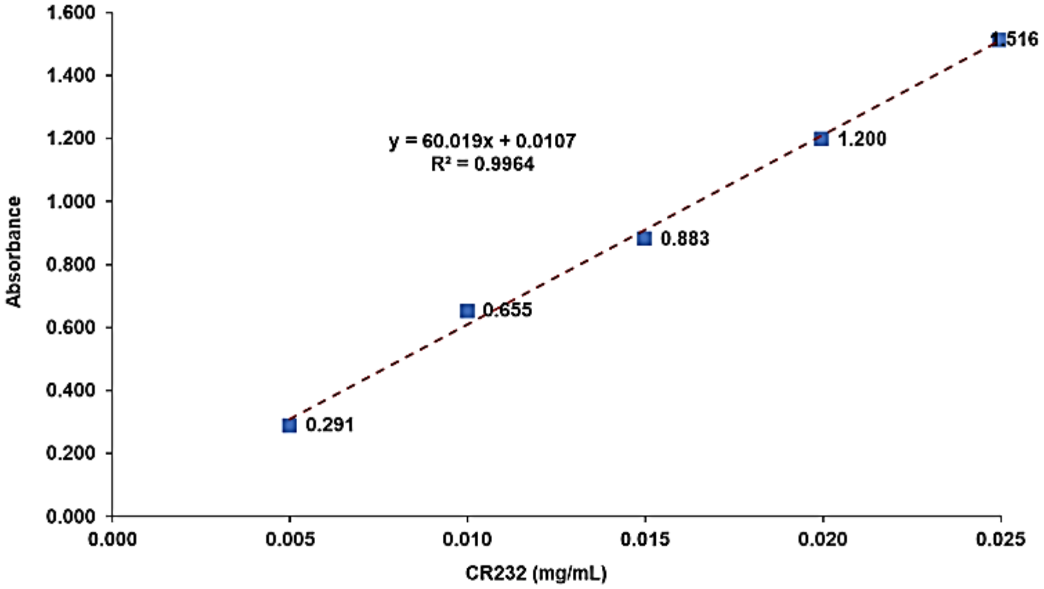

Figure S2. CR232 linear calibration model.

S.1.3.2. Determination of CR232 Content in the CR232-G5K NPs, DL% and EE%

6.0 mg of CR232-G5K were dissolved in 50 mL of DMSO obtaining a 0.120 mg/mL solution, which was analysed as such and then furtherly diluted to obtain CR232-G5K clear solutions at different concentrations (60, 30, 24 and 12 µg/mL), which were vigorously stirred for ten minutes to promote the release of CR232. The UV-Vis spectrum of each solution showed a new peak of absorbance at λ_{abs} = 328 nm corresponding to that obtained for the CR232-G5K NPs and a negligible peak of absorbance at λ_{abs} = 384 nm establishing that in DMSO the release of CR232 from the dendrimer NPs was insignificant. The amount of CR232 in the samples of CR232-G5K NPs

was consequently quantified by using the absorbance values at $λ_{abs}$ = 328 nm and Eq. (1). The samples of CR232-G5K NPs were analysed against a blank solution of the empty dendrimer G5K. Once estimated the content of CR232 in CR232-G5K NPs the values of DL% and EE% of CR232-G5K NPs were calculated from the following equations Eq. (2) and Eq. (3).

$$DL\ (\%) = \frac{\text{weight of the drug in NPs}}{\text{weight of the NPs}} \times 100 \quad (2)$$

$$EE\ (\%) = \frac{\text{weight of the drug in NPs}}{\text{inizial amount of drug}} \times 100 \quad (3)$$

Table S2 collects the results concerning the above-mentioned determinations.

Table S2. Values of A obtained for the five concentrations of CR232-G5K NPs analysed and the related C_{CR232} obtained from Eq. (1). Results concerning the concentration of CR232 in CR232-G5K NPs, DL%, EE%, molecular formula and MW of CR232-loaded NPs, as well as the difference between the MW obtained by ^{1}H NMR and that computed using UV-Vis, expressed as error %.

CR232-G5K NPs						
**A (mAU)**	**C_{CR232} (µg/mL)**	**CR232 in CR232-G5K**	**DL (%) EE (%)**	**Molecular Formula**	**MW**	**Error (%)**
2.35350	0.03903					
1.18140	0.01948	1.9121 ± 0.0389 mg*				
0.57283	0.009366	39.01± 0.7987mg§	31.7±0.6 98.3±2.0	$C_{1705}H_{2512}N_{398}O_{460}Cl_{233}$	44219.5±237.8 [1] 44153.1 [2]	0.15 [3]
0.54493	0.008901	41.2±0.7#				
0.33907	0.005482					

* mg of CR232 in 6.0 mg of CR232-G5K; § mg of CR232 in the obtained CR232-G5K NPs (123.2 mg); # moles of CR232 loaded per G5K mole; [1] computed by UV-Vis quantitative results; [2] by ^{1}H NMR; [3] computed on the mean.

S1.4. CR232-G5K NPs Molecular Weight (MW)

According to a previously reported procedure [3], the MW of CR232-G5K NPs was estimated both by its ^{1}H NMR spectrum and by the results of UV-Vis analyses, obtaining findings with a minimal difference (0.15%) (Table S2).

S1.5 Water Solubility of CR232, CR232-G5K NPs, and of the Nanotechnologically Manipulated CR232 Released in Water

The water solubility of the untreated CR232 pyrazole derivative, of CR232-G5K NPs and of nano-manipulated free CR232 contained in G5K NPs was determined performing the shake-flask method [2,4,5]. Excesses of CR232 (4.5 mg) and of CR232-G5K (6.1 mg) were added with water m-Q (2 mL and 1 mL respectively) obtaining suspensions which were incubated at 37 °C and stirred vigorously, observing (mainly for the free CR232) abundant foaming (pH = 7.4). The suspensions were maintained under stirring to promote the achievement of an equilibrium between the saturated solutions and the undissolved samples. Then, the suspensions were centrifugated (15 minutes, 3350 rpm) to precipitate the not solubilized material, which was separated by the supernatant solutions. The pale-yellow solid residues were washed several times with acetone to eliminate the residual water and brought to constant weight at reduced pressure. The supernatant solutions obtained were filtered using a 0.22 µm filter, and after having observed drops of the solutions with a Leica Galen III Professional Microscopes (Taylor Scientific, St. Louis, MO, USA) without detecting precipitate or differences with a drop of pure water, they were evaporated at reduced pressure and brought to constant weight under high vacuum obtaining the fractions of samples which were water-soluble (0.0045 ± 0.0002 mg for CR232 and 5.2 ± 0.05 mg in the case of CR232-G5K NPs). The amounts of the separated insoluble residues were 4.50 ± 0.03 mg for CR232 and 0.91 ± 0.02 mg for the CR232-G5K NPs, thus confirming the reliability of the weights obtained for the soluble solids, with errors of 0.1% and 1% respectively. The experiments were made in triplicate and the

solubilities of untreated CR232, of CR232-G5K NPs and of CR232 contained in G5K NPs were reported as mean ± SD. Table S3 reports the results obtained.

Table S3. Results obtained from solubility experiments performed on untreated CR232, CR232-G5K NPs.

Experimental Data		Solubility Data	Water-solubility	CR232 water solubility
Substance	mg	(mg/mL)	improvement	(mg/mL)
CR232-G5K NPs	5.2±0.05 [1]	5.2±0.05	2311.1	0.00225±0.0001
CR232 contained in solubilized CR232-G5K	1.65±0.02 [2]	1.65±0.02	733.3	

[1] mg of CR232-G5K NPs solubilized in water; [2] mg of CR232 contained in the solubilized NPs by DL% value.

S1.6. Dynamic Light Scattering (DLS) Analysis

Particle size (in nm), polydispersity index (PDI) and zeta potential (ζ-p) (mV) of G5K, CR232-G5K NPs and of CR232-SUVs were measured at 25 °C, at a scattering angle of 90° in m-Q water by using a Malvern Nano ZS90 light scattering apparatus (Malvern Instruments Ltd., Worcestershire, UK). Solutions of samples in m-Q water were diluted to final concentrations to have 250-600 kcps. The ζ-p value of all samples was recorded with the same apparatus. The results from these experiments were presented as the mean of three different determinations ± SD. Concerning the particle size distribution, intensity-based results were reported.

Table S4 collects the results obtained from DLS analyses on G5K and on CR232-G5K NPs concerning their size (Z-ave, nm), polydispersity index (PDI) and Zeta potential (ζ-p).

Table S4. Results obtained from DLS analyses on G5K NPs and CR232-G5K NPs: particle size (Z-ave, nm), PDI and ζ-p.

Measure	G5K NPs	CR232-G5K NPs
Z-Ave [1] (nm)	175.7±1.8	529.7±33.5
PDI	0.129±0.035	0.472±0.054
ζ-p (mV)	+48.0±6.4	+37.2±7.0

[1] Z-Ave = hydrodynamic diameter; ζ-p = Zeta potential.

S.1.7. Potentiometric Titration of G4K and BBB4-G4K NPs

Potentiometric titrations were performed at room temperature to construct the titration curves of G5K and CR232-G5K NPs and obtain the corresponding first derivatives. The samples (20-30 mg) were dissolved in 30 mL of Milli-Q water (m-Q) and treated with a standard 0.1 N NaOH aqueous solution [1.5 mL, pH = 10.20 (G5K) and 10.50 (CR232-G5K)]. The solutions were potentiometrically titrated by adding 0.2 mL aliquots of a standard 0.1 N HCl aqueous solution, up to total 3.0 mL and measuring the corresponding pH values [6]. Titrations were made in triplicate and the determinations were reported as mean ± SD (Figure S3).

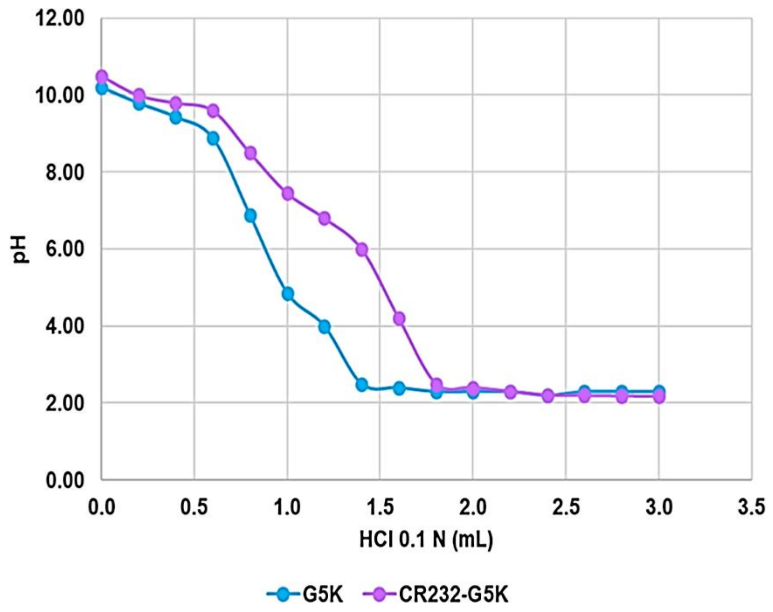

(**a**)

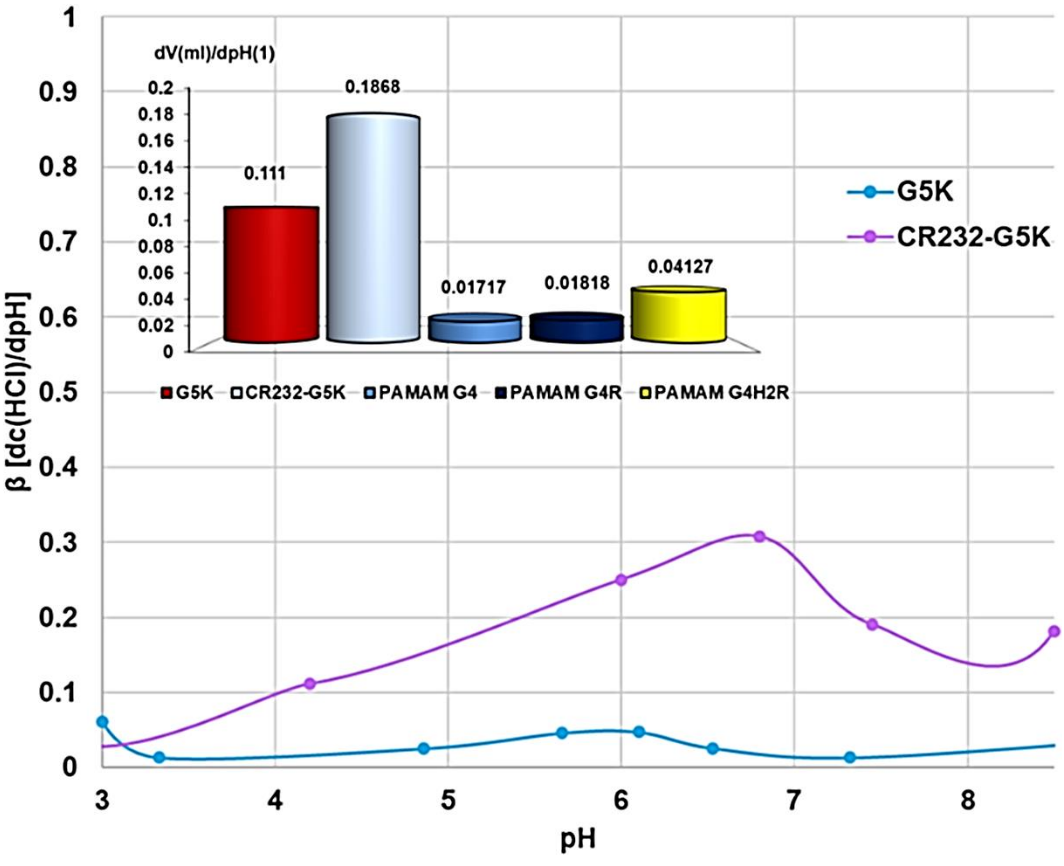

(**b**)

Figure S3. Titration curves (error bars not reported since difficult to detect) (**a**); β values vs. pH values and values of β mean (presented as bars graph) of CR232-G5K NPs and of three PAMAM of fourth generation for comparison.

S1.8. In Vitro CR232 Release Profile from CR232-G5K NPs

The release profile of CR232 from CR232-G5K NPs was investigated in vitro performing the dialysis bag diffusion method. Notably, 5.0 mg of NPs exactly weighted were dissolved in 2 mL of 0.1 M phosphate-buffered

saline (PBS, pH = 7.4), which should assure the dissolution of the complex according to the results obtained from water solubility determinations. Additionally, corresponding to the determined DL%, the sample should contain 1.583 mg of CR232. The solution was then positioned into a pre-swelled T2 tubular cellulose dialysis bag (flat width = 10 mm, wall thickness = 28 μm, V/cm = 0.32 mL, Membrane Filtration Products, Inc., Seguin, TX, USA) with a nominal molecular weight cut-off (MWCO) of 6000-8000 Da, bathed into 20 mL of 0.1 M PBS (pH 7.4, 37 °C), and gently stirred for 24 h. At predetermined time intervals (0 h, 1 h, 2 h, 3 h, 4 h, 5 h, 6 h, 8 h, 10 h, 12 h, 24 h), 10 mL were withdrawn from the incubation medium, were evaporated at reduced pressure, and were brought to constant weight at high vacuum, obtaining ten yellow solid residues. In a second moment, the residues were re-dissolved in DMSO, opportunely diluted with the same solvent to allows the quantification of the exact amount of CR232 present in the samples by using the UV-Vis apparatus and Eq. (1). The absorbance values (A) measurements were made at 384 nm in triplicate, and the results were reported as the mean ± SD of the three determinations. After sampling, an equal volume of fresh PBS was immediately replaced into the incubation medium.

The concentrations of CR232 released from CR232-G5K NPs were expressed as a cumulative release percentage (CR%) of the total amount of CR232 loaded in the CR232-G4K NPs in respect of the DL% value determined. To obtain the CR232 release profile from the dendrimer-based NPs, the CR232 CR% were plotted vs. times in a dispersion graph (Figure S4).

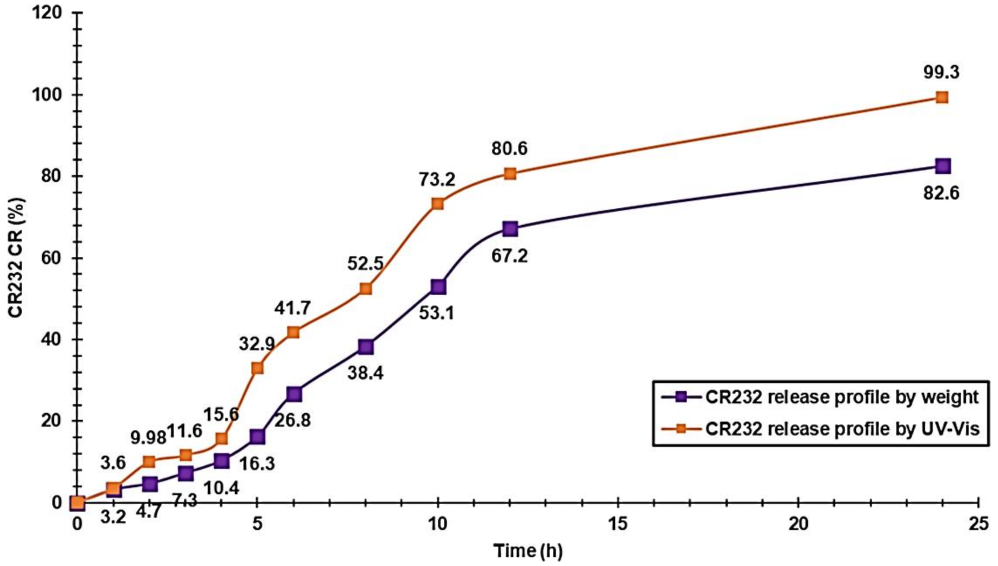

Figure S4. CR232 CR % at pH 7.4 for 24 hours obtained both by weighting the CR232 amounts passed in PBS solutions at fixed time points (purple line) and by UV-Vis analyses on these samples re-dissolved in DMSO (orange line). The error bars have not been reported on the graph to avoid confusion.

To determine the kinetics of the CR232 release and to investigate the main mechanisms which govern the release of CR232 from CR232-G5K NPs, we used different mathematical models, based on different mathematical functions, aimed at describing the drugs dissolution profiles. To select the most suitable function and to determine the most suitable mathematical kinetic model describing the dissolution profile of CR232, we fitted the CR% curve data with the zero order model (which reports in graph the % cumulative drug release vs. time), first-order model (Ln % cumulative drug remaining vs. time), Hixson-Crowell model (cube root of % cumulative drug remaining vs. time), Higuchi model (% cumulative drug release vs. square root of time), Korsmeyer-Peppas model (Ln % cumulative drug release vs. Ln of time) and Weibull model which reports in graph the values of LnLn(100/100-CR%) vs. the Ln of times values. A dispersion graph, whose linear regression proved a significantly higher value of R^2 (0.9754) was obtained by fitting the Weibull model, thus establishing that the CR232 release from the CR232-G5K NPs best fitted with the Weibull kinetic (Figure S5).

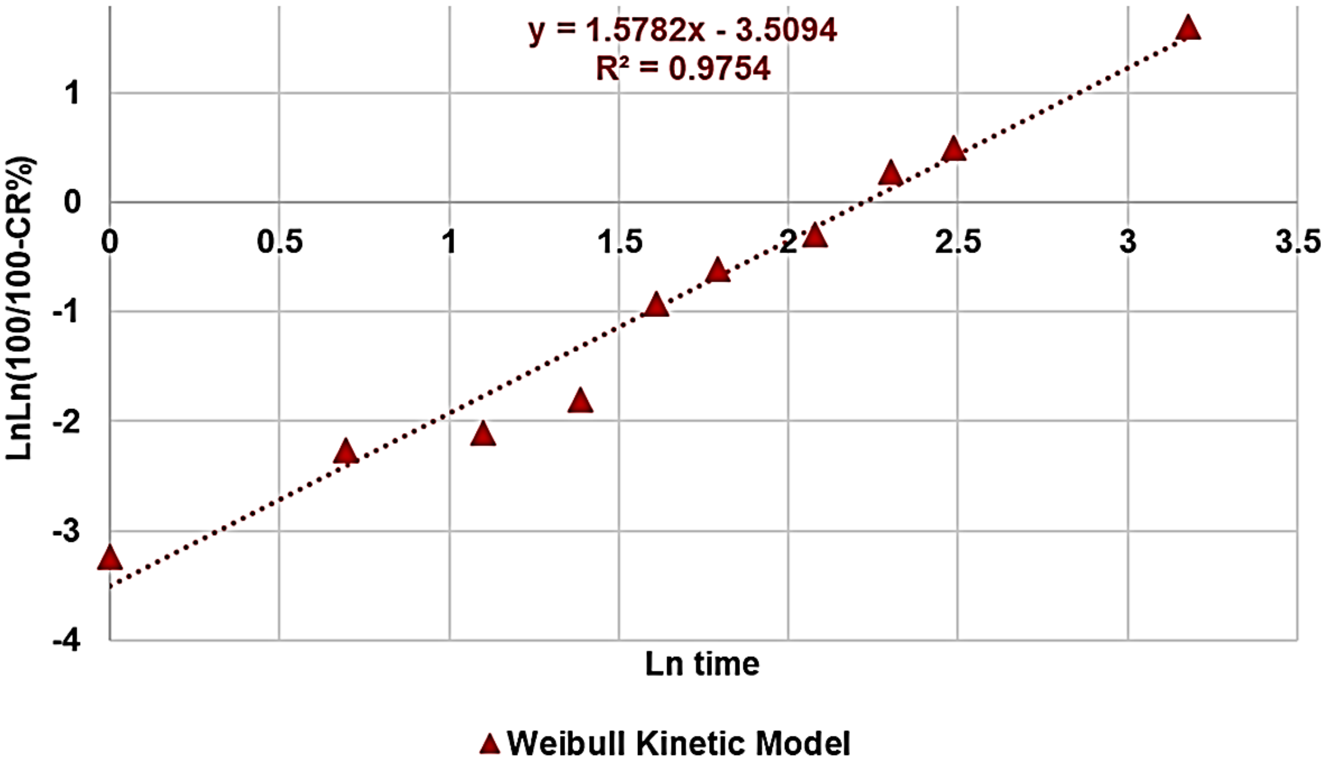

Figure S5. Linear regression of Weibull kinetic mathematical model with the related equation and R^2 value.

Section S2. Biological Investigations.

S2.1. Cytotoxicity Studies Performed for G5K on HeLa Cells

HeLa cell line, Dulbecco's Modified Eagle Medium (DMEM), Fetal Bovine Serum (FBS, 10%), non-essential amino acids, antibiotics (penicillin and streptomycin) and 3-(4,5-dimethylthiazol-2-yl)-2,5-diphenyl-2H-tetrazolium bromide (MTT) were purchased by Termofischer Scientific (Rodano, Milan, Italy). Dose-dependent in vitro experiments were performed for the reservoir dendrimer G5K to investigate its cytotoxic effects on HeLa cells. Briefly, HeLa cells were increased in DMEM enriched with FBS, 10%, non-essential amino acids (1%) and antibiotics (1%, penicillin and streptomycin) and maintained in an atmosphere containing 5% CO_2 at 37 °C. The cells were seeded at the density of 2 × 104 cells per well in a 24-well plate and in 4-wells slides in 500 µL of medium and incubated at 37 °C for 72 h. Subsequently, the cells were incubated with increasing concentrations (1–100 µM) of G5K at 37 °C for 24 h. Then 10 µL MTT was added into each well and after 4 h, the medium and MTT were discarded and 100 µL DMSO was added into each well. Finally, optical density at 490 nm was measured on a Termofischer Scientific microplate reader (Rodano, Milan, Italy) to determine cells viability (%). Commercial fourth generation amine-terminated polyamidoamine dendrimer (G4-PAMAM-NH_2) purchased by Merck (formerly Sigma-Aldrich, Darmstadt, Germany), having MW = 14214, was essayed in the same conditions as a positive control. Determinations were made in triplicate and results were expressed as mean percentage of the control (untreated cells) ± SD (Figure S6, Figure S7 and Table S5).

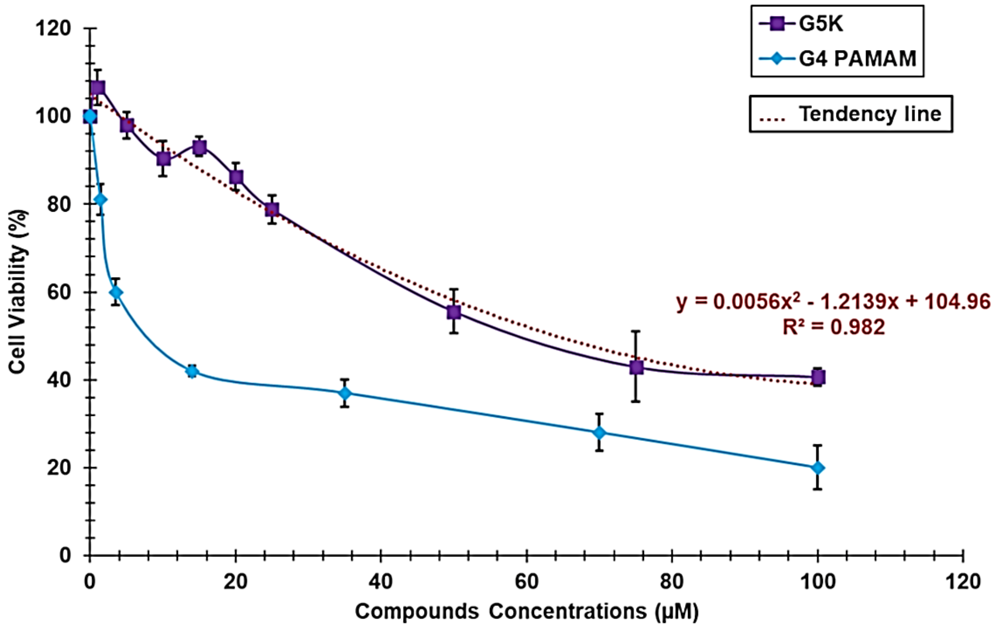

Figure S6. Cells viability (%) of HeLa cells exposed for 24 h to G5K and G4-PAMAM-NH_2 0–100 μM and the polynomial tendency line associated to the curve obtained for G5K with the equation Eq. (6) used to determine the LD_{50} of G5K.

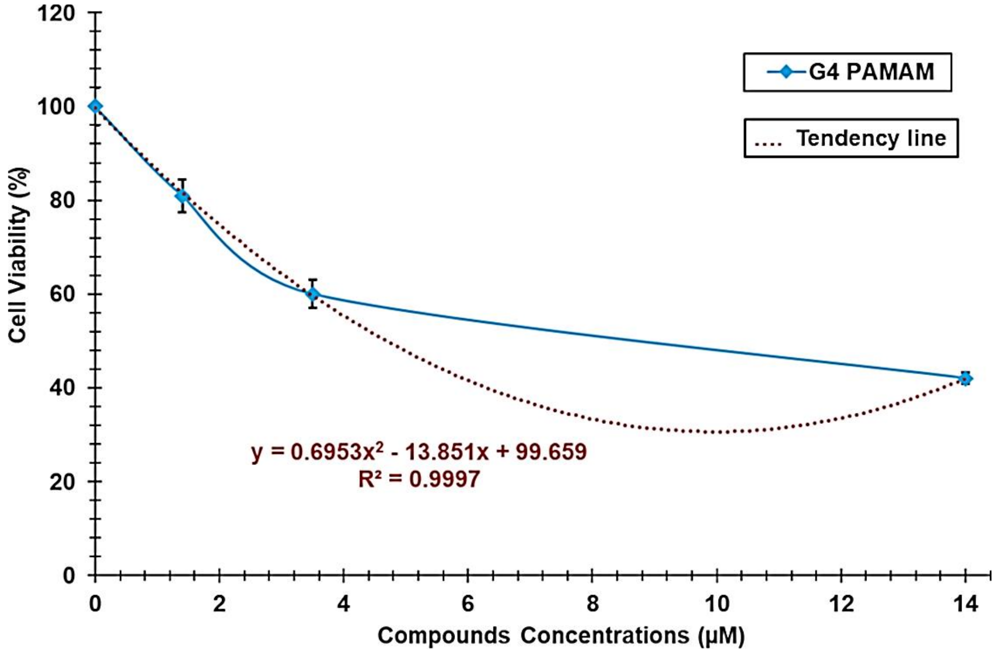

Figure S7. Cells viability (%) of HeLa cells exposed for 24 h to G4-PAMAM-NH_2 0–14 μM and the associated polynomial tendency line with the equation Eq. (7) used to determine the LD_{50}.

Table S5. Equations Eq. (6) and Eq. (7) and the LD_{50} values of G5K and of G4-PAMAM-NH_2 on HeLa cells (24h).

Dendrimer	Equations	R^2	LD_{50} (μM)
G5K	$y = 0.0056x^2$-1.2139x+104.96 (6)	0.9820	64.4
G4-PAMAM-NH_2	$y = 0.6953x^2$-13.851x+99.659 (7)	0.9997	4.7

S2.2. Dose and Time-Dependent Cytotoxicity Studies Performed for G5K, CR232 and CR232-G5K NPs on HaCaT Cells

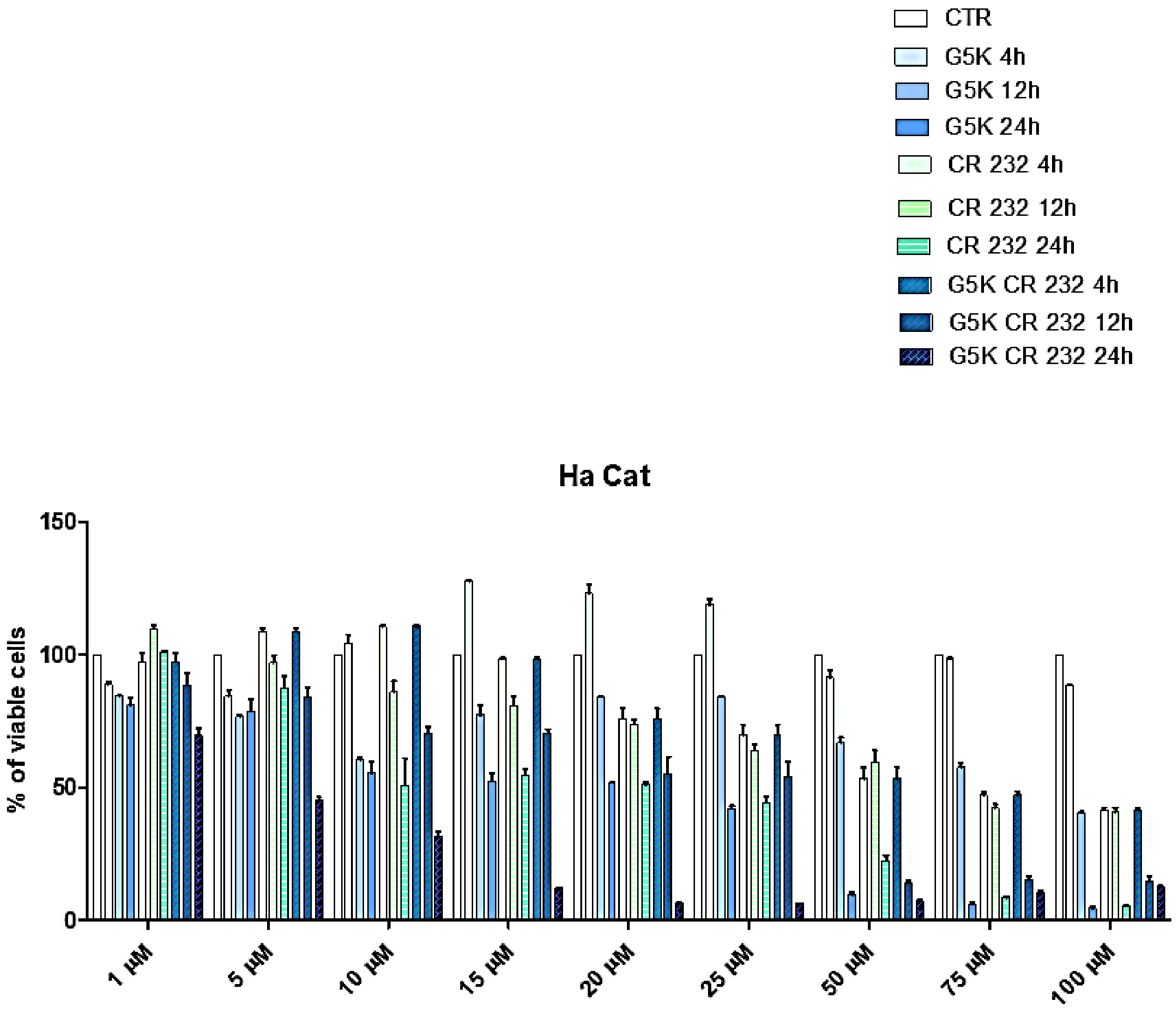

Figure S8. Dose- and time-dependent cytotoxicity activity of G5K, CR232 and CR232-G5K NPs at 4 h, 12 h and 24 h towards HaCaT cells.

Table S6. Results from statistical analysis. The statistical significance of differences between experimental and control groups was determined via a two-way analysis of variance (ANOVA) with the Bonferroni correction.

Parameter				
Table Analyzed	G5K CR232			
Two-way ANOVA				
Source of Variation	% of total variation	P value		
Interaction	16.77	< 0.0001		
Column Factor	52.55	< 0.0001		
Row Factor	29.54	< 0.0001		
Source of Variation	P value summary	Significant?		
Interaction	***	Yes		
Column Factor	***	Yes		

Row Factor	***	Yes		
Source of Variation	Df	Sum-of-squares	Mean square	F
Interaction	72	50489	701.2	36.52
Column Factor	9	158231	17581	915.6
Row Factor	8	88942	11118	579.0
Residual	180	3456	19.20	
Number of missing values	0			
Bonferroni posttests				
CTR vs G5K 4h				
Row Factor	CTR	G5K 4h	Difference	95% CI of diff.
1	100.0	88.89	-11.11	-23.58 to 1.365
5	100.0	84.52	-15.48	-27.95 to -3.005
10	100.0	104.4	4.409	-8.061 to 16.88
15	100.0	127.7	27.69	15.22 to 40.16
20	100.0	123.1	23.07	10.60 to 35.54
25	100.0	118.9	18.91	6.438 to 31.38
50	100.0	91.51	-8.494	-20.96 to 3.977
75	100.0	98.28	-1.720	-14.19 to 10.75
100	100.0	88.53	-11.47	-23.94 to 0.9971
Row Factor	Difference	t	P value	Summary
1	-11.11	3.104	P < 0.05	*
5	-15.48	4.325	P<0.001	***
10	4.409	1.232	P > 0.05	ns
15	27.69	7.741	P<0.001	***
20	23.07	6.447	P<0.001	***
25	18.91	5.285	P<0.001	***
50	-8.494	2.374	P > 0.05	ns
75	-1.720	0.4807	P > 0.05	ns
100	-11.47	3.207	P < 0.05	*
CTR vs G5K 12h				
Row Factor	CTR	G5K 12h	Difference	95% CI of diff.
1	100.0	84.53	-15.47	-27.94 to -3.003
5	100.0	76.73	-23.27	-35.74 to -10.80
10	100.0	60.52	-39.48	-51.95 to -27.01
15	100.0	77.33	-22.67	-35.14 to -10.20
20	100.0	84.07	-15.93	-28.40 to -3.464
25	100.0	84.09	-15.91	-28.38 to -3.441
50	100.0	67.00	-33.00	-45.47 to -20.53
75	100.0	57.71	-42.29	-54.76 to -29.82
100	100.0	40.42	-59.58	-72.05 to -47.11
Row Factor	Difference	t	P value	Summary

1	-15.47	4.325	P<0.001	***
5	-23.27	6.504	P<0.001	***
10	-39.48	11.03	P<0.001	***
15	-22.67	6.335	P<0.001	***
20	-15.93	4.454	P<0.001	***
25	-15.91	4.447	P<0.001	***
50	-33.00	9.224	P<0.001	***
75	-42.29	11.82	P<0.001	***
100	-59.58	16.65	P<0.001	***
CTR vs G5K 24h				
Row Factor	CTR	G5K 24h	Difference	95% CI of diff.
1	100.0	81.06	-18.94	-31.41 to -6.466
5	100.0	78.79	-21.21	-33.68 to -8.738
10	100.0	55.60	-44.40	-56.87 to -31.93
15	100.0	52.62	-47.38	-59.85 to -34.91
20	100.0	51.85	-48.15	-60.62 to -35.68
25	100.0	42.09	-57.91	-70.38 to -45.44
50	100.0	9.723	-90.28	-102.7 to -77.81
75	100.0	6.058	-93.94	-106.4 to -81.47
100	100.0	4.649	-95.35	-107.8 to -82.88
Row Factor	Difference	t	P value	Summary
1	-18.94	5.293	P<0.001	***
5	-21.21	5.928	P<0.001	***
10	-44.40	12.41	P<0.001	***
15	-47.38	13.24	P<0.001	***
20	-48.15	13.46	P<0.001	***
25	-57.91	16.19	P<0.001	***
50	-90.28	25.23	P<0.001	***
75	-93.94	26.26	P<0.001	***
100	-95.35	26.65	P<0.001	***
CTR vs CR 232 4h				
Row Factor	CTR	CR 232 4h	Difference	95% CI of diff.
1	100.0	97.39	-2.613	-15.08 to 9.857
5	100.0	108.7	8.704	-3.766 to 21.17
10	100.0	110.8	10.79	-1.684 to 23.26
15	100.0	98.37	-1.633	-14.10 to 10.84
20	100.0	75.90	-24.10	-36.57 to -11.63
25	100.0	69.79	-30.21	-42.68 to -17.74
50	100.0	53.39	-46.61	-59.09 to -34.14
75	100.0	47.21	-52.79	-65.26 to -40.32
100	100.0	41.51	-58.49	-70.96 to -46.02
Row Factor	Difference	t	P value	Summary
1	-2.613	0.7305	P > 0.05	ns
5	8.704	2.433	P > 0.05	ns

10	10.79	3.015	P < 0.05	*
15	-1.633	0.4564	P > 0.05	ns
20	-24.10	6.736	P<0.001	***
25	-30.21	8.444	P<0.001	***
50	-46.61	13.03	P<0.001	***
75	-52.79	14.75	P<0.001	***
100	-58.49	16.35	P<0.001	***
CTR vs CR 232 12h				
Row Factor	CTR	CR 232 12h	Difference	95% CI of diff.
1	100.0	109.9	9.893	-2.577 to 22.36
5	100.0	96.93	-3.069	-15.54 to 9.401
10	100.0	86.04	-13.96	-26.43 to -1.494
15	100.0	80.87	-19.13	-31.60 to -6.655
20	100.0	73.71	-26.29	-38.76 to -13.82
25	100.0	63.83	-36.17	-48.64 to -23.70
50	100.0	59.66	-40.34	-52.81 to -27.87
75	100.0	42.49	-57.51	-69.98 to -45.04
100	100.0	40.82	-59.18	-71.65 to -46.71
Row Factor	Difference	t	P value	Summary
1	9.893	2.765	P > 0.05	ns
5	-3.069	0.8578	P > 0.05	ns
10	-13.96	3.903	P<0.01	**
15	-19.13	5.346	P<0.001	***
20	-26.29	7.347	P<0.001	***
25	-36.17	10.11	P<0.001	***
50	-40.34	11.28	P<0.001	***
75	-57.51	16.07	P<0.001	***
100	-59.18	16.54	P<0.001	***
CTR vs CR 232 24h				
Row Factor	CTR	CR 232 24h	Difference	95% CI of diff.
1	100.0	101.0	1.017	-11.45 to 13.49
5	100.0	87.42	-12.58	-25.05 to -0.1107
10	100.0	50.89	-49.11	-61.58 to -36.64
15	100.0	54.67	-45.33	-57.80 to -32.86
20	100.0	51.15	-48.85	-61.32 to -36.38
25	100.0	44.20	-55.80	-68.27 to -43.33
50	100.0	22.51	-77.49	-89.96 to -65.02
75	100.0	8.685	-91.31	-103.8 to -78.84
100	100.0	5.527	-94.47	-106.9 to -82.00
Row Factor	Difference	t	P value	Summary
1	1.017	0.2843	P > 0.05	ns
5	-12.58	3.516	P<0.01	**
10	-49.11	13.73	P<0.001	***
15	-45.33	12.67	P<0.001	***

20	-48.85	13.65	P<0.001	***
25	-55.80	15.60	P<0.001	***
50	-77.49	21.66	P<0.001	***
75	-91.31	25.52	P<0.001	***
100	-94.47	26.40	P<0.001	***
CTR vs G5K CR 232 4h				
Row Factor	CTR	G5K CR 232 4h	Difference	95% CI of diff.
1	100.0	97.39	-2.613	-15.08 to 9.857
5	100.0	108.7	8.704	-3.766 to 21.17
10	100.0	110.8	10.79	-1.684 to 23.26
15	100.0	98.37	-1.633	-14.10 to 10.84
20	100.0	75.90	-24.10	-36.57 to -11.63
25	100.0	69.79	-30.21	-42.68 to -17.74
50	100.0	53.39	-46.61	-59.09 to -34.14
75	100.0	47.21	-52.79	-65.26 to -40.32
100	100.0	41.51	-58.49	-70.96 to -46.02
Row Factor	Difference	t	P value	Summary
1	-2.613	0.7305	P > 0.05	ns
5	8.704	2.433	P > 0.05	ns
10	10.79	3.015	P < 0.05	*
15	-1.633	0.4564	P > 0.05	ns
20	-24.10	6.736	P<0.001	***
25	-30.21	8.444	P<0.001	***
50	-46.61	13.03	P<0.001	***
75	-52.79	14.75	P<0.001	***
100	-58.49	16.35	P<0.001	***
CTR vs G5K CR 232 12h				
Row Factor	CTR	G5K CR 232 12h	Difference	95% CI of diff.
1	100.0	88.49	-11.51	-23.98 to 0.9566
5	100.0	84.06	-15.94	-28.41 to -3.473
10	100.0	70.45	-29.55	-42.02 to -17.08
15	100.0	70.54	-29.46	-41.93 to -16.99
20	100.0	55.12	-44.88	-57.35 to -32.41
25	100.0	54.05	-45.95	-58.42 to -33.48
50	100.0	14.24	-85.76	-98.23 to -73.29
75	100.0	15.44	-84.56	-97.03 to -72.09
100	100.0	14.86	-85.14	-97.61 to -72.67
Row Factor	Difference	t	P value	Summary
1	-11.51	3.218	P < 0.05	*
5	-15.94	4.456	P<0.001	***
10	-29.55	8.259	P<0.001	***
15	-29.46	8.235	P<0.001	***
20	-44.88	12.54	P<0.001	***
25	-45.95	12.84	P<0.001	***

50	-85.76	23.97	P<0.001	***
75	-84.56	23.63	P<0.001	***
100	-85.14	23.80	P<0.001	***
CTR vs G5K CR 232 24h				
Row Factor	CTR	G5K CR 232 24h	Difference	95% CI of diff.
1	100.0	69.54	-30.46	-42.93 to -17.99
5	100.0	45.30	-54.70	-67.17 to -42.23
10	100.0	31.91	-68.09	-80.56 to -55.62
15	100.0	12.18	-87.82	-100.3 to -75.35
20	100.0	6.449	-93.55	-106.0 to -81.08
25	100.0	6.488	-93.51	-106.0 to -81.04
50	100.0	7.333	-92.67	-105.1 to -80.20
75	100.0	10.25	-89.75	-102.2 to -77.28
100	100.0	12.94	-87.07	-99.54 to -74.59
Row Factor	Difference	t	P value	Summary
1	-30.46	8.514	P<0.001	***
5	-54.70	15.29	P<0.001	***
10	-68.09	19.03	P<0.001	***
15	-87.82	24.55	P<0.001	***
20	-93.55	26.15	P<0.001	***
25	-93.51	26.14	P<0.001	***
50	-92.67	25.90	P<0.001	***
75	-89.75	25.09	P<0.001	***
100	-87.07	24.33	P<0.001	***

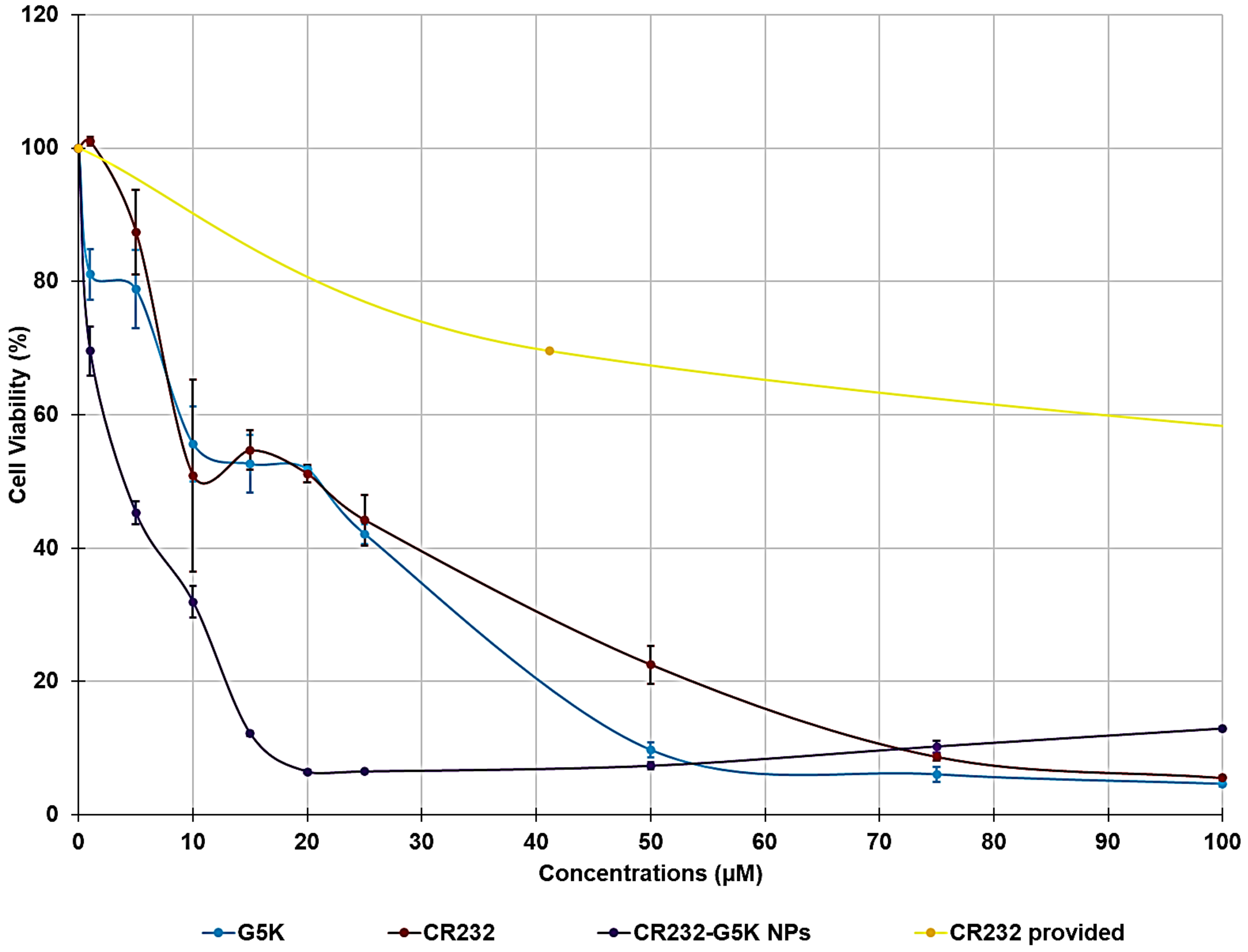

Figure S9. Dose-depended cytotoxicity curves obtained reporting in graph the cell viability % vs. the concentrations of G5K, CR232, CR232-G5K NPs at 24 hours of exposition and the amount of nanoengineered CR232 provided by the quantity of CR232-G5K NPs administered

References

1. Alfei, S.; Spallarossa, A.; Lusardi, M.; Zuccari, G. Successful Dendrimer and Liposome-Based Strategies to Solubilize an Antiproliferative Pyrazole Otherwise Not Clinically Applicable. *Nanomaterials* **2022**, *12*, 233. https://doi.org/10.3390/nano12020233.
2. Zuccari, G.; Alfei, S.; Zorzoli, A.; Marimpietri, D.; Turrini, F.; Baldassari, S.; Marchitto, L.; Caviglioli, G. Resveratrol-loaded D-tocopheryl polyethylene glycol 1000 succinate micelles as nutritional supplement for children with chronic liver disease. *Pharmaceutics* **2021**, *13*, 1128.
3. Alfei, S.; Schito, A.M.; Zuccari, G. Considerable Improvement of Ursolic Acid Water Solubility by Its Encapsulation in Dendrimer Nanoparticles: Design, Synthesis and Physicochemical Characterization. *Nanomaterials* **2021**, *11*, 2196.
4. Alfei, S.; Brullo, C.; Caviglia, D.; Zuccari, G. Preparation and Physicochemical Characterization of Water-Soluble Pyrazole-Based Nanoparticles by Dendrimer Encapsulation of an Insoluble Bioactive Pyrazole Derivative. *Nanomaterials* **2021**, *11*, 2662.
5. Zuccari, G.; Baldassari, S.; Alfei, S.; Marengo, B.; Valenti, G.E.; Domenicotti, C.; Ailuno, G.; Villa, C.; Marchitto, L.; Caviglioli, G. D-α-Tocopherol-Based Micelles for Successful Encapsulation of Retinoic Acid. *Pharmaceuticals* **2021**, *14*, 212.
6. Benns, J. M.; Choi, J. S.; Mahato, R. I.; Park, J. S.; Kim, S. W. pH-sensitive cationic polymer gene delivery vehicle: N-Ac-poly(L-histidine)-graft-poly(L-lysine) comb shaped polymer. *Bioconj. Chem.* **2000**, *11*, 637-645.
